# Kinetic Cytokine Secretion Profile of LPS-Induced Inflammation in the Human Skin Organ Culture

**DOI:** 10.3390/pharmaceutics12040299

**Published:** 2020-03-25

**Authors:** Raanan Gvirtz, Navit Ogen-Shtern, Guy Cohen

**Affiliations:** The Skin Research Institute, The Dead-Sea and Arava Science Center, Masada 86910, Israel; raanan@adssc.org (R.G.); navit@adssc.org (N.O.-S.)

**Keywords:** drug development, biological biomarkers of skin inflammation, cytokine, ex vivo, human skin organ culture, LPS

## Abstract

Several in vitro models that mimic different aspects of local skin inflammation exist. The use of ex vivo human skin organ culture (HSOC) has been reported previously. However, comprehensive evaluation of the cytokine secretory capacity of the system and its kinetics has not been performed. Objective: the aim of the current study was to investigate the levels and secretion pattern of key cytokine from human skin tissue upon lipopolysaccharide (LPS) stimulation. HSOC maintained in an air–liquid interface was used. Epidermal and tissue viability was monitored by MTT and Lactate Dehydrogenase (LDH) activity assay, respectively. Cytokine levels were examined by ELISA and multiplex array. HSOCs were treated without or with three different LPS subtypes and the impact on IL-6 and IL-8 secretion was evaluated. The compounds enhanced the secreted levels of both cytokines. However, differences were observed in their efficacy and potency. Next, a kinetic multiplex analysis was performed on LPS-stimulated explants taken from three different donors to evaluate the cytokine secretion pattern during 0–72 h post-induction. The results revealed that the pro-inflammatory cytokines IL-6, IL-8, TNFα and IL-1β were up-regulated by LPS stimuli. IL-10, an anti-inflammatory cytokine, was also induced by LPS, but exhibited a different secretion pattern, peak time and maximal stimulation values. IL-1α and IL-15 showed donor-specific changes. Lastly, dexamethasone attenuated cytokine secretion in five independent repetitions, supporting the ability of the system to be used for drug screening. The collective results demonstrate that several cytokines can be used as valid inflammatory markers, regardless of changes in the secretion levels due to donor’s specific alterations.

## 1. Introduction

The skin is the largest organ of the human body, primarily acting to maintain homeostasis and protect the body from the deleterious action of the environment. Apart of its vital functions, the skin serves an important role in the defense mechanism against pathogens and possesses immunomodulatory properties [1]. Upon external or internal signals, skin-resident cells, such as Langerhans cells, keratinocytes, melanocytes, mast cells and macrophages, secrete small, hormone-like signal peptides called cytokines that act as local immune modulators or recruit additional immune cells [2]. Their action depends on the presence of specific membrane receptors found on the majority of cells. Though inflammation is a common feature of several skin diseases including psoriasis, atopic dermatitis, seborrheic dermatitis and contact dermatitis [3], the characteristics of the cellular immune response and composition of cytokine profile vary among them [4]. Several in vitro and in vivo models mimic the inflammatory response of the skin [5,6,7,8,9]. Three main in vitro platforms are available: cell cultures (e.g., keratinocyte, Langerhans cells, co-cultures, etc.), 3D reconstructed skin equivalent and human/porcine organ culture (ex vivo models). Cell cultures, stimulated by lipopolysaccharide (LPS) or 12-O-tetradecanoylphorbol-13-acetate (TPA), are routinely-used methods for both gaining basic knowledge of the molecular pathways and regulation of the immune response, and as models for evaluating anti-inflammatory properties of novel agents [10,11]. Commercial 3D-reconstructed skin equivalents are well established and validated models. Still, they suffer from high costs and flexibility that prevents them from being used more often [12]. The usage of ex vivo organ cultures for evaluations is of great interest as, hypothetically, it can mimic the normal local response and the complex interactions between the cellular residents of the epidermis and dermis. Although several excellent studies used this system [13,14], the kinetics and profile of the secreted cytokines are not fully elucidated. Thus, the objective of the current study was to monitor the secreted profile of 15 key cytokines in response to LPS and to assess the benefits and limitations of this experimental system.

## 2. Materials and Methods

All cell culture media and reagents were purchased from biological industries Ltd. (Beit-HaEmek, Israel). Lipopolysaccharide (LPS), Dexamethasone, and chemical reagents were purchased from Sigma-Aldrich, Rehovot, Israel.

### 2.1. Human Skin Organ Cultures

The skin tissues were obtained from 37–65-year-old healthy women undergoing aesthetic abdomen surgery, after signing an informed consent form. All experiments were conducted with the approval of the IRB (Helsinki Committee) of Soroka Medical Center, Beer Sheva, Israel. The experiments were initiated at the day of the surgery. Skin culture preparation and treatments were performed under aseptic conditions. A mechanical skin press apparatus was used to section the skin to 0.8 × 0.8 cm^2^ pieces, as previously described [15]. The skin explants were maintained in an air-liquid interface, dermal side submerged in the medium, as described before [16]. Serum-free Dulbecco’s Modified Eagle Medium (DMEM, 265 mg/L calcium), supplemented with 100 μg/mL penicillin and 100 μg/mL streptomycin, were used.

### 2.2. Determination of Epidermal and Organ Culture Viability

Following LPS treatment, the epidermis was separated from dermis, and viability was determined, as described before [17]. Briefly, the skin was incubated for 1 min in phosphate-buffered saline (PBS) at 56 °C, after which the epidermis was physically detached from the dermis using forceps and scalpel. The epidermis pieces were then placed in a 96-well plate and incubated with 0.5 mg/mL MTT (3-(4,5-Dimethylthiazol-2-yl)-2,5-diphenyltetrazolium bromide) at 37 °C for 1 h. The samples were then transferred to a new 96-well plate, and isopropanol was added to solubilize the dye. The absorbance was measured in an ELISA reader. In addition, the viability of the skin organ culture was determined by the Lactate Dehydrogenase (LDH Activity Assay Kit; Sigma Aldrich). In this method, used in several skin studies [18,19], tissue damage will increase LDH release into the media and will result in increased conversion of NAD to NADH, which is specifically detected by colorimetric (450 nm) assay.

### 2.3. Cytokine Quantification

Following treatments, the spent media of the skin organ culture was collected and centrifuged, and the clear supernatant was stored at −80 °C until analyzed. Cytokine levels (IL-6 and IL-8) were evaluated by commercial ELISA, according to the manufacturer’s instructions (Biolegend, San Diego, CA, USA). All other cytokine levels were determined by multiplex analysis (Quansys Biosciences, London, UK).

### 2.4. Statistical Analysis

Results are given as mean ± SEM. Statistical analyses were performed using student’s *t*-test (GraphPad Prism). P < 0.05 was considered significant.

## 3. Results

**Figure 1:**
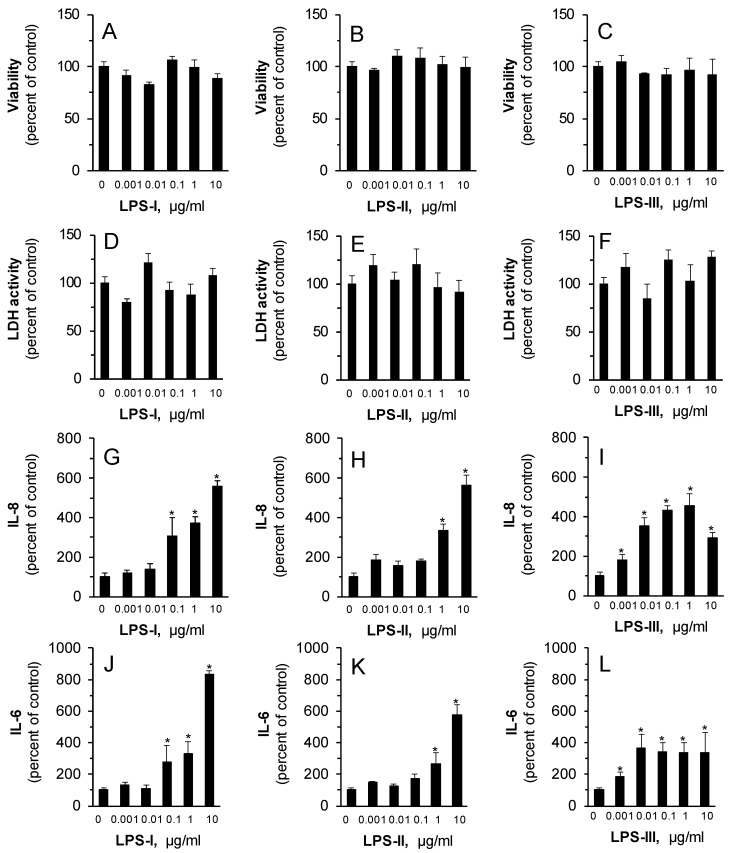
Lipopolysaccharide (LPS) enhances cytokine secretion in human skin organ culture in a dose-dependent manner. The skin explants were treated without or with increasing concentrations of LPS ((1) E.coli O111:B4; (2) Salmonella enterica serotype enteritidis; (3) Salmonella enterica serotype typhimurium) for 48 hrs. **A**–**C**: The epidermal layer was taken for viability test using the MTT assay. **D**–**F** skin viability was determined by Lactate Dehydrogenase (LDH) assay. **G**–**I** and **J**–**L**: the secreted levels of IL-8 and IL-6 from the human skin organ culture were quantified by ELISA. Results are shown in MEAN ± SEM, *n* = 3. * P < 0.05 for difference from the untreated control group.

The impact of three types of LPS ((1) E.coli O111:B4; (2) Salmonella enterica serotype enteritidis; (3) Salmonella enterica serotype typhimurium) was evaluated ex vivo, in the human skin organ culture system (HSOC). The skin explants were treated without or with increasing concentrations of LPS for 48 h following incubation, and the epidermal viability and IL-6 and IL-8 secreted levels were evaluated, as written in the Materials and Methods section. As shown in Figure 1 A–C, all compounds were well tolerated by the skin explants and showed no toxic effect. In addition, to exclude dermal damage, lactate dehydrogenase activity assay was performed. The results confirmed that all LPS preparations were well tolerated by the organ culture (Figure 1D–F). Importantly, all LPS subtypes tested enhanced IL-6 and IL-8 secretion in a dose-dependent manner. Although LPS-III showed the highest potency, LPS-I had the highest efficacy, resulting in increased levels of IL-6 and IL-8 by 8.3- and 5.5-fold, respectively.

**Figure 2:**
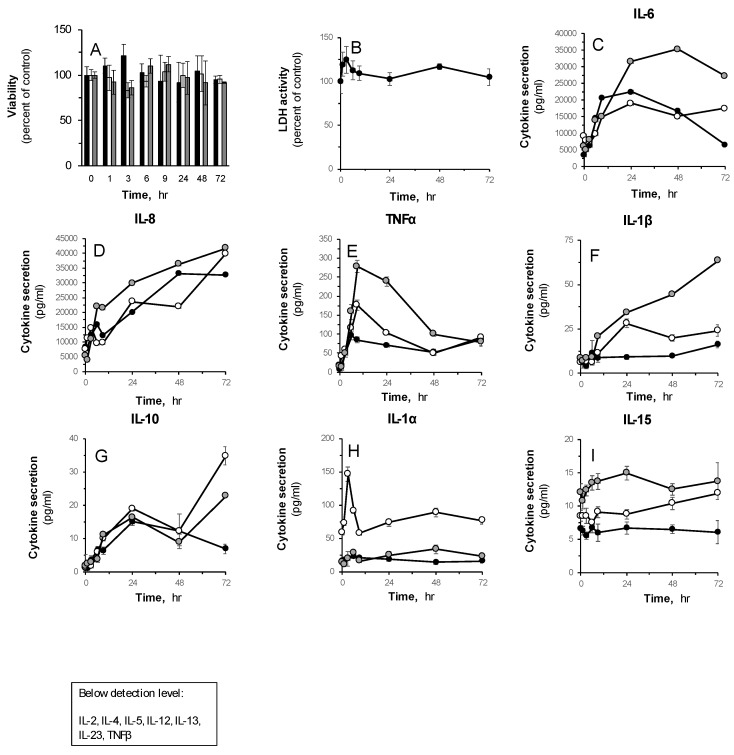
Time-dependent impact of LPS on cytokine secretion levels in the human skin organ culture. The skin explants were treated without or with LPS ((1) E.coli O111:B4; 5 µg/mL) at the indicated time points. **A**: The epidermal layer was taken for viability test by MTT assay. **B**: The viability of the skin organ culture was determined by LDH. **C**–**I**: the secreted cytokine levels in the human skin organ culture were quantified by multiplex. Each curve represents a different donor. Box: specified cytokines’ amounts were below detection levels. Results are presented as MEAN ± SEM, *n* = 3. Average skin weight was 155 mg ± 4.3, without any impact of the treatment.

Next, a time-course analysis was performed upon treatment with LPS-I (5 µg/mL). The kinetic secretion profiles of IL-6, IL-8, TNFα, IL-1β, IL-10, IL-1α and IL-15 over 72 h are shown in Figure 2. The skin organ remained vital throughout the treatment (Figure 2A,B). Of note, the levels of IL-2, IL-4, IL-5, IL-12, IL-13, IL-23 and TNFβ were below the detection level in these experimental parameters. One can observe that the basal levels of IL-6 and IL-8 are predominant in the HSOC system. Upon LPS stimulation, IL-6 reached its highest levels within 24 h (Figure 2, upper-right panel), whereas IL-8 levels increased rapidly during the first 6 h and continued to accumulate for up to 72 h, the maximum tested duration. The secreted levels of TNFα peaked at 9 h, and then declined over time. Still, they remained elevated in comparison to the untreated control group also after 72 h. It should be emphasized that although the basal levels of TNFα were similar in all three donors, they differed greatly upon stimulation. Nonetheless, the dynamic reaction to LPS showed similar patterns. This phenomenon was also observed in the case of IL-1β, which despite differences among donors, showed consistent increases in cytokine levels throughout the experiment. The levels of IL-10 showed a time-dependent elevation at 24 h. After 72 h of incubation with LPS, a significant discrepancy was observed between replications, making it difficult to relate to the nature of the cytokine dynamics. The secretion of IL-15 and IL-1α were unaltered in this experimental system, excluding a rapid and massive accumulation of IL-1α in a single skin donor (6 h, Figure 2, lower panel).

**Figure 3:**
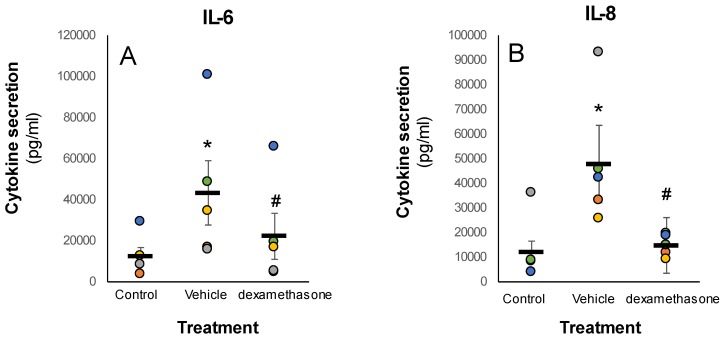
Dexamethasone attenuated LPS-induced cytokine secretion in the human skin organ culture. The skin explants were treated without or with LPS for 48 h (5 µg/mL). The secreted levels of IL-6 (**A**) and IL-8 (**B**) in the human skin organ culture were quantified by ELISA. Results are shown as MEAN ± SEM, *n* = 5. *^/#^ P < 0.05 for difference from the untreated control group or LPS-stimulated (vehicle) group, respectively.

Lastly, the impact of Dexamethasone on IL-6 and IL-8 secretion levels in stimulated skin explants was evaluated. Dexamethasone was added concomitantly with LPS. After 48 h, the secreted levels of both cytokines were monitored. As expected, IL-6 and IL-8 levels were enhanced by the LPS stimuli, resulting in approximately 3.5- and 3.9-fold increase, respectively (Figure 3). Importantly, dexamethasone effectively attenuated the hypersecretion of both cytokines, which strengthens the validity of the experimental system.

## 4. Discussion

The aim of the study was to gain insight into the local inflammatory response of the skin tissue upon external stimulation. Overall, the results of the multiplex cytokine analysis demonstrate that several cytokines can be used as reliable and valid inflammatory markers, regardless of donor-dependent variabilities and the amounts secreted. The use of the HSOC system for investigating the complex reaction of the skin upon induction of inflammation, and as a platform for drug discovery, derives from its advantages as a full-thickness, human-derived model, and its direct correlation to clinical outcomes, including donor (patient)-specific response [20]. In addition to LPS-induced inflammation systems, the ex vivo skin explants can also be used to monitor and screen different aspects of cutaneous disorders, including UVB-induced damage, oxidative stress, skin irritation and functional assays [5,13,21,22]. Moreover, by using different inflammatory stimuli, such as IL-17 and TNFα instead of LPS, the HSOC can be used as a model for specific skin disorders, such as psoriasis [11,14,23]. However, the use of the ex vivo system has its disadvantages. The complex multicellular interactions may lead to unclear results, and the inherent differences among donors and the lack of a reservoir of immune cells to be recruited following chemokine and cytokine secretion, challenge the uses of the method. In addition, the diversity of maintenance conditions for the explants, such as sera, media and growth supplements, may lead to variations in the results and cytokine production [24]. Thus, the materials and sources of any study should be clearly stated and standardized in the future.

Cytokines are small secreted proteins that serve as key modulators of the innate immune system and allow homeostasis and routine function of the different cell types composing the skin [25]. Their action depends on specific receptor-mediated signaling pathways, of which the JAK-STAT and NF-kB are the two main signal transduction pathways activated in inflammatory skin disorders [4]. The secretion profile and kinetics depend on both cell-specific expressions of inflammatory mediators and the original signaling that caused their secretion. In the current study, we have evaluated the inflammatory reaction to LPS stimulation. Lipopolysaccharide (LPS) is a well-known pathogen-associated molecular pattern, primarily found on the outer leaflet of the external membrane in most gram-negative bacteria [26]. Although the LPS molecule varies among different organisms, especially in the length and fatty acid composition of the lipid A domain, the basic structure is mostly preserved [27]. Here we show that the efficacy and potency of three selected LPS types (E.coli O111:B4; Salmonella enterica serotype enteritidis; Salmonella enterica serotype typhimurium) in inducing cytokine secretion varies. This may also account for some of the variability of results in other studies [14,16,20,28]. Thus, in order to increase the reproducibility among researchers and get additional insights into results, the specific origin and supplier of LPS should be clearly stated. It should be noted that response to LPS does not necessarily predict host response to infection, but is rather a simplified experimental system.

LPS induces its main effect by activation of the Toll-like receptor (TLR) family members, which are predominant pattern-recognition receptors [29]. To date, more than ten sub-types are known, of which the majority are expressed on immune and non-immune skin-cells surfaces [30,31]. The current study demonstrates the existence of variable secretion profiles among the different cytokines following inflammation induction. This may be the result of more than one TLR receptor subtype’s activation [32]. Alternatively, cytokines themselves are known to modulate paracrine secretion by creating negative or positive feedbacks in reaction to their expression. For instance, elevated levels of the anti-inflammatory cytokine IL-10 may lead to the restriction of additional inflammatory agents by the IL-10/STAT3 pathway [33,34], whereas elevated amounts of TNFα could promote the secretion of other cytokines by the TNFR1 pathway [35]. IL-6 and IL-8 levels were found to be highly abundant in the ex vivo system. Both their basal and stimulated levels were the highest found and therefore are suggested for routine use in anti-inflammatory screenings. Intriguingly, their kinetics differ, as IL-6 levels reach a steady state after shorter period of time. In addition, TNFα and IL-1β showed a high reaction to LPS and can be used as additional inflammatory markers.

Among the cytokines tested in the multiplex analysis presented in Figure 2, several were below the detection levels. IL-4, IL-13 and IL-5 are T helper-2 (Th-2)-derived cytokines, predominantly secreted by T cells, but also by mast cells, basophils, and eosinophils [4,36]. These cells may be detected in samples taken from the peripheral skin, though in low concentration, which may be the cause for misdetection. IL-2 is also secreted by activated T cells, though of the Th-1 branch of the immune response [4]. Interestingly, IL-15 and IL-10, which are secreted by T helper cells as well, are also produced by keratinocytes and dendritic cells in the skin [4], and are accordingly detected in the activated spent media (Figure 2). It should be noted that IL-2 and IL-5 were reportedly detected, at the transcriptome level, in keratinocytes from healthy and lupus human skin in the minority of individuals [37]. Such publications, however, are rare. IL-12 and IL-23 are mostly secreted by antigen-presenting cells (APCs) [38]. IL-12 was found to be upregulated in human dendritic cells upon LPS-stimulation [39], as well as in the skin-resident Langerhans cells (LCs), although in much lower amounts compared to secretion in monocyte-derived LCs [40]. IL-23 is also secreted by dendritic cells and macrophages [41]. Both cytokines were found to be expressed in vitro in very low amounts in healthy human keratinocytes at the transcriptome and proteome levels. Moreover, the induction of their secretion is attributed to immune-mediated inflammation, such as occurs in psoriatic lesions, rather than LPS stimulation or bacterial-infection [42,43], which may also explain the lack of their detection in the HSOC. Reports regarding TNF beta, alternatively symbolled Lymphotoxin-α, being expressed in human skin residential cells are very rare. Some report the existence of its mRNA in keratinocyte cells, and upregulation upon fungal infections [44] and Malassezia yeasts [44], though not at the protein level, which supports the difficulty found in detecting its secretion by the model.

To conclude, the use of the ex vivo system for anti-inflammatory screening and for the investigation of the complex interplay between the regulatory mechanisms of cytokine secretion is valid. This study also demonstrates that when using a particular pro-inflammatory or anti-inflammatory marker, it is necessary to measure its amounts when kinetically suitable, meaning after sufficient accumulation of the cytokine but prior to its degradation. The results show that each cytokine has its unique kinetic secretion profile and that the experimental parameters chosen for each cytokine should be properly adjusted. Additional studies should be performed, including on in vitro–in vivo correlation, to fully understand the response of the skin to pathogens and the limitation of the LPS-induced skin inflammation system.

## References

[1] Pasparakis M., Haase I., Nestle F.O. (2014). Mechanisms regulating skin immunity and inflammation. Nat. Rev. Immunol..

[2] Nedoszytko B., Sokołowska-Wojdyło M., Ruckemann-Dziurdzińska K., Roszkiewicz J., Nowicki R.J. (2014). Chemokines and cytokines network in the pathogenesis of the inflammatory skin diseases: Atopic dermatitis, psoriasis and skin mastocytosis. Postep. Dermatologii i Alergol..

[3] Proksch E., Brandner J.M., Jensen J.M. (2008). The skin: An indispensable barrier. Exp. Dermatol..

[4] Coondoo A. (2011). Cytokines in dermatology—A basic overview. Indian J. Dermatol..

[5] Andrade T.A., Aguiar A.F., Guedes F.A., Leite M.N., Caetano G.F., Coelho E.B., Das P.K., Frade M.A. (2015). Ex vivo model of human skin (hOSEC) as alternative to animal use for cosmetic tests. Procedia Eng..

[6] Semlin L., Schäfer-Korting M., Borelli C., Korting H.C. (2011). In vitro models for human skin disease. Drug Discov. Today.

[7] Mathes S.H., Ruffner H., Graf-Hausner U. (2014). The use of skin models in drug development. Adv. Drug Deliv. Rev..

[8] Corzo-León D.E., Munro C.A., MacCallum D.M. (2019). An ex vivo human skin model to study superficial fungal infections. Front. Microbiol..

[9] Maboni G., Davenport R., Sessford K., Baiker K., Jensen T.K., Blanchard A.M., Wattegedera S., Entrican G., Tötemeyer S. (2017). A novel 3D skin explant model to study anaerobic bacterial infection. Front. Cell. Infect. Microbiol..

[10] Park K., Lee J.-H., Cho H.-C., Cho S.-Y., Cho J.-W. (2010). Down-regulation of IL-6, IL-8, TNF-α and IL-1β by glucosamine in HaCaT cells, but not in the presence of TNF-α. Oncol. Lett..

[11] Balato A., Lembo S., Mattii M., Schiattarella M., Marino R., De Paulis A., Balato N., Ayala F. (2012). IL-33 is secreted by psoriatic keratinocytes and induces pro-inflammatory cytokines via keratinocyte and mast cell activation. Exp. Dermatol..

[12] Netzlaff F., Lehr C.M., Wertz P.W., Schaefer U.F. (2005). The human epidermis models EpiSkin®, SkinEthic® and EpiDerm®: An evaluation of morphology and their suitability for testing phototoxicity, irritancy, corrosivity, and substance transport. Eur. J. Pharm. Biopharm..

[13] Portugal-Cohen M., Soroka Y., Frušić-Zlotkin M., Verkhovsky L., Brégégère F.M., Neuman R., Kohen R., Milner Y. (2011). Skin organ culture as a model to study oxidative stress, inflammation and structural alterations associated with UVB-induced photodamage. Exp. Dermatol..

[14] Yehuda H., Soroka Y., Zlotkin-Frušić M., Gilhar A., Milner Y., Tamir S. (2012). Isothiocyanates inhibit psoriasis-related proinflammatory factors in human skin. Inflamm. Res..

[15] Wineman E., Douglas I., Wineman V., Sharova K., Jaspars M., Meshner S., Bentwich Z., Cohen G., Shtevi A. (2015). Commiphora gileadensis sap extract induces cell cycle-dependent death in immortalized keratinocytes and human dermoid carcinoma cells. J. Herb. Med..

[16] Kahremany S., Babaev I., Gvirtz R., Ogen-Stern N., Azoulay-Ginsburg S., Senderowitz H., Cohen G., Gruzman A. (2019). Nrf2 Activation by SK-119 Attenuates Oxidative Stress, UVB, and LPS-Induced Damage. Skin Pharmacol. Physiol..

[17] Ogen-Shtern N., Chumin K., Cohen G., Borkow G. (2019). Increased pro-collagen 1, elastin, and TGF-β1 expression by copper ions in an ex-vivo human skin model. J. Cosmet. Dermatol..

[18] Hodgkinson T., Bayat A. (2016). In vitro and ex vivo analysis of hyaluronan supplementation of integra® dermal template on human dermal fibroblasts and keratinocytes. J. Appl. Biomater. Funct. Mater..

[19] Messager S., Hann A.C., Goddard P.A., Dettmar P.W., Maillard J.Y. (2003). Assessment of skin viability: Is it necessary to use different methodologies?. Ski. Res. Technol..

[20] Portugal-Cohen M., Ish-Shalom E., Mallon R., Corral P., Michoux F., Ma’or Z. (2018). Apple of Sodom (Calatropis procera) Callus Extract, a Novel Skincare Active and Its Biological Activity in Skin Models When Combined with Dead Sea Water. J. Cosmet. Dermatol. Sci. Appl..

[21] Ng K.W., Pearton M., Coulman S., Anstey A., Gateley C., Morrissey A., Allender C., Birchall J. (2009). Development of an ex vivo human skin model for intradermal vaccination: Tissue viability and Langerhans cell behaviour. Vaccine.

[22] Hanson K.M., Clegg R.M. (2002). Observation and quantification of ultraviolet-induced reactive oxygen species in Ex Vivo human skin. Photochem. Photobiol..

[23] Guilloteau K., Paris I., Pedretti N., Boniface K., Juchaux F., Huguier V., Guillet G., Bernard F.X., Lecron J.C., Morel F. (2010). Skin inflammation induced by the synergistic action of IL-17A, IL-22, Oncostatin M, IL-1, and TNF-Recapitulates some features of psoriasis. J. Immunol..

[24] Coolen N.A., Vlig M., Van Den Bogaerdt A.J., Middelkoop E., Ulrich M.M.W. (2008). Development of an in vitro burn wound model. Wound Repair Regen..

[25] Bak R.O., Mikkelsen J.G. (2010). Regulation of cytokines by small RNAs during skin inflammation. J. Biomed. Sci..

[26] La Vecchia C., Garattini S. (2002). An ideal minister of health. J. Epidemiol. Community Health.

[27] Helen F., Kanchana G. (2014). Investigation on the properties of L-serine doped zinc tris (thiourea) sulphate crystal for NLO application. Indian J. Pure Appl. Phys..

[28] Companjen A.R., Van Der Wel L.I., Wei L., Laman J.D., Prens E.P. (2001). A modifiedex vivo skin organ culture system for functional studies. Arch. Dermatol. Res..

[29] Newton K., Dixit V.M. (2012). Signaling in innate immunity and inflammation. Cold Spring Harb. Perspect. Biol..

[30] Portou M.J., Baker D., Abraham D., Tsui J. (2015). The innate immune system, toll-like receptors and dermal wound healing: A review. Vascul. Pharmacol..

[31] Miller L.S. (2008). Toll-like receptors in skin. Adv. Dermatol..

[32] Good D.W., George T., Watts B.A. (2012). Toll-like receptor 2 is required for LPS-induced toll-like receptor 4 signaling and inhibition of ion transport in renal thick ascending limb. J. Biol. Chem..

[33] Couper K.N., Blount D.G., Riley E.M. (2008). IL-10: The Master Regulator of Immunity to Infection. J. Immunol..

[34] Hutchins A.P., Diez D., Miranda-Saavedra D. (2013). The IL-10/STAT3-mediated anti-inflammatory response: Recent developments and future challenges. Brief. Funct. Genomics.

[35] Banno T., Gazel A., Blumenberg M. (2004). Effects of tumor necrosis factor-α (TNFα) in epidermal keratinocytes revealed using global transcriptional profiling. J. Biol. Chem..

[36] Wills-Karp M., Finkelman F.D. (2008). Untangling the complex web of IL-4-and IL-13-mediated signaling pathways. Sci. Signal..

[37] Carneiro J.R.M., Fuzii H.T., Kayser C., Alberto F.L., Soares F.A., Sato E.I., Andrade L.E.C. (2011). IL-2, IL-5, TNF-α and IFN-γ mRNA expression in epidermal keratinocytes of systemic lupus erythematosus skin lesions. Clinics.

[38] Teng M.W.L., Bowman E.P., McElwee J.J., Smyth M.J., Casanova J.L., Cooper A.M., Cua D.J. (2015). IL-12 and IL-23 cytokines: From discovery to targeted therapies for immune-mediated inflammatory diseases. Nat. Med..

[39] Kang J., Kim S.C., Han S.C., Hong H.J., Jeon Y.J., Kim B., Koh Y.S., Yoo E.S., Kang H.K. (2012). Hair-loss preventing effect of Grateloupia elliptica. Biomol. Ther..

[40] Peiser M., Wanner R., Kolde G. (2004). Human epidermal Langerhans cells differ from monocyte-derived Langerhans cells in CD80 expression and in secretion of IL-12 after CD40 cross-linking. J. Leukoc. Biol..

[41] Schirmer C., Klein C., Von Bergen M., Simon J.C., Saalbach A. (2010). Human fibroblasts support the expansion of IL-17-producing T cells via up-regulation of IL-23 production by dendritic cells. Blood.

[42] Larsen J.M., Bonefeld C.M., Poulsen S.S., Geisler C., Skov L. (2009). IL-23 and TH17-mediated inflammation in human allergic contact dermatitis. J. Allergy Clin. Immunol..

[43] Piskin G., Sylva-Steenland R.M.R., Bos J.D., Teunissen M.B.M. (2006). In Vitro and In Situ Expression of IL-23 by Keratinocytes in Healthy Skin and Psoriasis Lesions: Enhanced Expression in Psoriatic Skin. J. Immunol..

[44] Shiraki Y., Ishibashi Y., Hiruma M., Nishikawa A., Ikeda S. (2006). Cytokine secretion profiles of human keratinocytes during Trichophyton tonsurans and Arthroderma benhamiae infections. J. Med. Microbiol..

